# Effect of Preoperative Music Therapy Versus Intravenous Midazolam on Anxiety, Sedation and Stress in Stomatology Surgery: A Randomized Controlled Study

**DOI:** 10.3390/jcm12093215

**Published:** 2023-04-29

**Authors:** Filippo Giordano, Mariateresa Giglio, Irene Sorrentino, Fabio Dell’Olio, Pantaleo Lorusso, Maria Massaro, Angela Tempesta, Luisa Limongelli, Laura Selicato, Gianfranco Favia, Giustino Varrassi, Filomena Puntillo

**Affiliations:** 1 Department of Precision and Regenerative Medicine and Ionian Area, Aldo Moro University, 70124 Bari, Italy; filippo.giordano@uniba.it (F.G.); irene.sorrentino@gmail.com (I.S.); lorussopantaleo@gmail.com (P.L.); brimas08@gmail.com (M.M.); 2 Department of Interdisciplinary Medicine, Aldo Moro University, 70124 Bari, Italy; gianfranco.favia@uniba.it (G.F.); f.delliolo.odo@outlook.it (F.D.); angelatempesta1989@gmail.com (A.T.); luisa.limongelli@gmail.com (L.L.); nucciapuntillo@gmail.com (F.P.); 3 Department of Mathematic, Aldo Moro University, 70124 Bari, Italy; 4 Department of Research, Paolo Procacci Foundation, 00193 Roma, Italy; giuvarr@gmail.com

**Keywords:** music therapy, anxiety, stress, sedation, surgery

## Abstract

Background: Patients undergoing surgery and general anesthesia often experience anxiety, fear and stress, with negative bodily responses. These may be managed by the pre-procedural application of anxiolytic, analgesic, and anesthetic drugs that have, however, potential risks or side effects. Music therapy (MT) can be used as a complementary no-drug intervention alongside standard surgical care before, during and after medical procedures. The aim of this study was to evaluate the effects of preoperative MT intervention compared to premedication with midazolam on levels of anxiety, sedation and stress during general anesthesia for elective stomatology surgery. Methods: A two-arm randomized and controlled single-center, parallel-group, pre–post event study was conducted. In total, 70 patients affected by stage I or II (both clinically and instrumentally N0) micro-invasive oral cancer and undergoing elective surgery under general anesthesia were assigned to the control group (CG) or to the music therapy group (MTG). MTG patients received preoperative music therapy intervention (MT) from a certified music therapist before surgery, while the CG patients did not receive MT but instead received premedication with intravenous midazolam, 0.02 mg/kg. Anesthesia was the same in both groups. The systolic blood pressure (SBP), diastolic blood pressure (DBP) and heart rate (HR) were recorded at the entrance to the operating room, just before the induction of anesthesia and every 5 min until the end of surgery. An anxiety visual analogues scale (A-VAS) was used to evaluate the level of anxiety. The bispectral index (BIS) monitor was used to measure the depth of sedation just before and 10 min after both music intervention and midazolam administration. Stress response was assessed 5 min before and 20 min after surgery via the control of plasma prolactin (PRL), growth hormone (GH), and cortisol levels. The patient global impression of satisfaction (PGIS) was tested 1 h after surgery. Participants in the MTG were asked to answer 3 questions concerning their experience with MT. Results: No statistical differences among the PRL, GH and cortisol levels between the two groups were registered before and after the treatment, as well as for PAS, PAD and HR. Significant differences in the A-VAS scores between the MTG and CG (*p* < 0.01) was observed. Compared to the CG, MTG patients had a statistically significantly lower BIS score (*p* = 0.02) before induction. A PGIS score of 86.7% revealed that patients in the MTG were very satisfied, versus 80% in the CG (*p* < 0.05). Conclusion: Preoperative music therapy could be an alternative to intravenous midazolam when aiming to promote a preoperative and post-operative state of anxiolysis and sedation in stomatology surgery, even if no differences were found in terms of the surgery-related stress response according to physiological and hormonal determinations.

## 1. Introduction

Patients undergoing surgery and general anesthesia often experience anxiety, fear and stress, which generate negative bodily responses, such as tachycardia, hypertension, an increased myocardial consumption of O_2_, arrhythmias, increased peripheral resistance, hypercoagulability, immunodeficiency and catabolic response [1].

Emotional distress and pain may be managed by the pre-procedural application of anxiolytics and analgesics [2], which are disadvantaged by potential risks or side effects [3,4]. This may result in adverse outcomes, such as delayed healing, increased healthcare utilization, delirium and cost [5,6,7]. Furthermore, a recent Cochrane review showed that benzodiazepines reduce pre-procedural anxiety compared to placebos, but the evidence used to support this was of a low quality [8].

Music therapy (MT) is defined as the clinical and evidence-based use of music interventions by a trained professional in order accomplish individualized goals within a therapeutic relationship [9,10]. MT can be used as a safe and cost-effective complementary no-drug intervention [11,12,13], alongside standard surgical care [14], to prevent and treat emotional distress [15] and pain [16] before, during and after medical procedures [17,18].

Evidence from Cochrane systematic reviews shows that MT can decrease anxiety in patients with cancer [19], coronary heart diseases [20] and in patients on mechanical ventilation [9].

Some studies have reported that music interventions reduce sedative requirements in patients undergoing regional anesthesia when combined with sedation, using both midazolam [21,22] and propofol, and also in critically ill patients in intensive care units [9,13].

However, most of the previous studies have been performed using music medicine, which is characterized by passive listening to pre-recorded music that is selected and administered by medical personnel or non-music therapy staff [23,24,25].

Due to shortcomings regarding the methods used and quality control standards, results regarding the effects of MT versus benzodiazepine on anxiety, sedation and stress have been heterogeneous and poor.

No trials have been conducted using MT in stomatology surgery.

The present study was conducted in order to evaluate the effects of preoperative MT, compared to premedication with midazolam, in patients undergoing general anesthesia for elective stomatology surgery.

The first hypothesis was that a preoperative MT intervention can have the same effects as midazolam on anxiety, stress and sedation.

The secondary hypothesis was that MT can be described as a pleasant experience that allows for a better patient satisfaction than pre-anesthesia with midazolam.

## 2. Methods

### 2.1. Participants

This study was approved by the Hospital Ethics Committee of Bari (n° 6256) and written informed consent was obtained from all subjects participating in the trial. The trial was registered prior to patient enrollment at clinicaltrials.gov (accessed on 6 April 2023) under the reference ID NCT05417529 (https://clinicaltrials.gov/ct2/show/NCT05417529?term=music+therapy&cntry=IT&draw=2&rank=10, accessed on 29 September 2022).

This study was a two-arm randomized and controlled single-center, parallel-group, pre–post event study.

**Figure 1:**
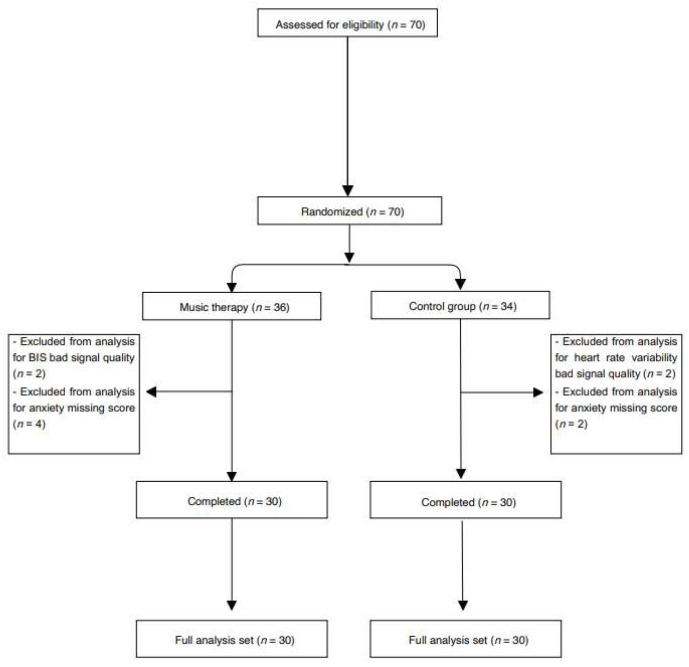
CONSORT flow chart.

All adult patients affected by stage I or II (both clinically and instrumentally N0) micro-invasive oral cancer, referred to the Stomatology Unit of the University Hospital of Bari, Italy, and receiving elective surgery under general anesthesia were screened for the study and randomized according to the study protocol (Figure 1).

The exclusion criteria were as follows: (a) <18 years; (b) with severe neurological or psychiatric conditions; (c) with hearing impairment; (d) with drug abuse problems; and (e) an American Society of Anesthesiologists (ASA) score of IV to V.

The study was conducted in accordance with the Helsinki declaration for human rights and adheres to the applicable CONSORT guidelines (Table S1).

### 2.2. Procedures

On the day of the surgery, participants were informed in both an oral and written manner that the data collected during the study would be recorded anonymously, and that their personal information would be kept confidential. All patients signed an informed consent form. Then, they were assigned to the control group (CG) or the MT group (MTG) by computer sample randomization. Patient randomization was carried out by one of the authors (F.D.), who the only one to have access to the sequence, using an online software version 1.4 (www.random.org, accessed on 29 September 2022).

The MTG patients did not receive premedication with intravenous midazolam and received the music therapy intervention from a certified music therapist (MTp) fellow of guided imagery and music.

MTp had never met the patients before the start of the study.

The music treatment consisted of 3 stages:

- Firstly, 30 min before surgery, at the bedside, the MTp engaged the patient in an individual brief conversation (10 min) in order to identify their preferred musical genre/songs. Based on both the information collected and specific music elements, the MTp prepared customized playlists to listen to that were tailored to the needs of each patient [26]. The MTp used music that was selected from classical music of the Western tradition, pop, rock, new age, soundtrack and light jazz. A patient-centered approach was employed. Using an interactive relational approach to receptive MT [27,28] that was supplemented by an adaptation of the Bonny method, namely Guided Imagery and Music in the medical setting [29], the MTp tailored the interventions to patients’ individual needs at the time of the surgery.
- After being monitored in the operating room, patients were prepared to listen to the music by the short guided relaxation of the breath (music was matched rhythmically to the rate of breathing, then gradually slowed to encourage slower, deeper breathing) [30], and were invited to find an image with a positive association to focus on. This step took approximately 2 min.
- Music listening, before anesthesia induction, lasted 5 min. Participants listened to music via noise-canceling headphones (BOSE^®^ quiet comfort 35 II) from an iPod^®^, and the volume was controlled by the MTp.

The CG patients did not receive the music therapy intervention and received only premedication with intravenous midazolam, 0.02 mg/kg.

All patients were monitored using standard methods (electrocardiogram, non-invasive blood pressure, pulse oximetry), and were also connected to a bispectral index (BIS) monitor to measure the depth of sedation.

Anesthesia was the same in both groups. Induction was carried out by fentanyl in the dose of 3 mcg/kg, propofol in the dose of 2 mg/kg and rocuronium in the dose of 1 mg/kg in order to facilitate intubation. Anesthesia was maintained with sevoflurane (1.5–2% end-tidal concentration) and was adjusted to maintain a bi-spectral index (BIS) value between 50 and 60. Intraoperative analgesia was obtained using fentanyl boluses of 1 mcg/kg as required. The neuromuscular blockade was monitored with a peripheral nerve stimulator and rocuronium doses were given accordingly.

### 2.3. Outcomes

Systolic blood pressure (SBP), diastolic blood pressure (DBP), heart rate (HR), peripheral nerve stimulation (PNS) and carbon dioxide (CO_2_) were recorded at the entrance to the operating room, just before the induction of anesthesia and every 5 min until the end of surgery.

The BIS score was recorded just before and 10 min after both the music intervention and midazolam administration. The BIS measures the effects of the sedatives on the brain: it displays a value below 40 when there is no cortical activity or the patient is in coma; a value between 40 and 60 when the patient is unconscious; a value between 70 and 90 when there are varying levels of conscious sedation; and a value of 100 when the patient is fully awake [31].

An anxiety visual analogues scale (A-VAS) [32] was used to evaluate the level of anxiety from 0 (no anxiety) to 10 (maximum anxiety) before the intervention (music or midazolam) and 1 h after the end of the surgical procedure. The stress response was assessed 5 min before and 20 min after surgery by controlling the plasma prolactin (PRL), growth hormone (GH), and cortisol levels.

The patient global impression of satisfaction (PGIS) was tested 1 h after surgery using a 4-item score (1 = very dissatisfied, 2 = dissatisfied, 3 = satisfied, 4 = very satisfied).

**Figure 2:**
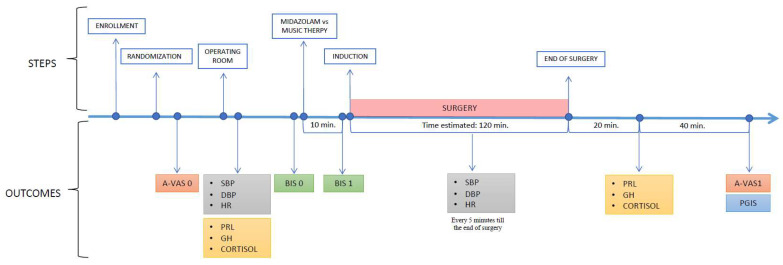
Outcomes timeline. A-VAS: anxiety visual analogue scale; SBP: systolic blood pressure; DPB: diastolic blood pressure; HR: heart rate; PRL: prolactin; GH: growth hormone; BIS: bispectral index; PGIS: patient global impressions scale.

The timing record of the outcomes is shown in Figure 2.

Data collection was carried out by a different researcher that was unaware of the group allocation the patients.

Three simple questions about the music experience were administered to the MTG patients only by one of the researchers: (1) Have you appreciated listening to the music? (2) Has music positively influenced your level of anxiety related to surgery? (3) Would you recommend someone undergoing surgery to listen to music? Five answers were possible for the first 2 questions: not at all, somewhat, moderately so, a lot and very much so. For the last question, the possible answers were yes, no, and I do not know.

### 2.4. Statistical Analysis

This study was a randomized and controlled trial using a two-arm, parallel-group, pre–post event, and mixed-methods approach to compare the effects of preoperative music listening on anxiety sedation and stress during stomatology surgery compared to premedication with midazolam.

Differences between the two groups were inspected for all the variables available. All variables were collected as mean and standard deviation, except for A-VAS and BIS, which were expressed as median. Continuous variables were examined according to a t-test or a Mann–Whitney statistical test according to normal data distribution. A two-tailed test was performed by default, which was integrated into a one-tailed test in the presence of difference between the groups, which was confirmed by the first test. Probability distributions were investigated using a Shapiro test. Differences between the two groups for the categorical variable were checked by Fisher’s exact test. All the tests were considered using a significance level of 0.05.

A power analysis was performed by considering 30 samples for each group with an effect size of 0.735, a significance level 0.05 and a power of 0.80; in addition, a sample estimation confirmed the number of samples used, with the same parameters.

## 3. Results

In total, 70 patients were enrolled (36 in the MTG and 34 in the CG). In 10 patients, the data were not complete, and they were excluded from the final analysis. Therefore, 30 patients were analyzed for each group. No differences were retrieved in terms of age, sex, ASA classification surgery type and duration of surgery (Table 1).

No statistical differences were registered in the PRL, GH and cortisol levels between the two groups before the interventions (i.e., music or midazolam administration), as well as for PAS, PAD, HR, BIS, and A-VAS Table 2 (a). Panel b of Table 2 shows the relative *p*-values for the variables after the interventions: no statistical differences were found between the two groups for all variables, except for BIS and A-VAS.

More specifically, the median value of the BIS was 98 in both groups before the interventions (*p* = 0.27), but it reduced to 89 in the MTG and 90 in the CG, *p* < 0.05).

In addition, A-VAS presents no differences between the two groups before the interventions (*p* = 0.67, median: 6 in both groups), whereas significant differences were found after the interventions (median: 0 in MTG vs. 2 in CG, *p* < 0.05).

**Figure 3:**
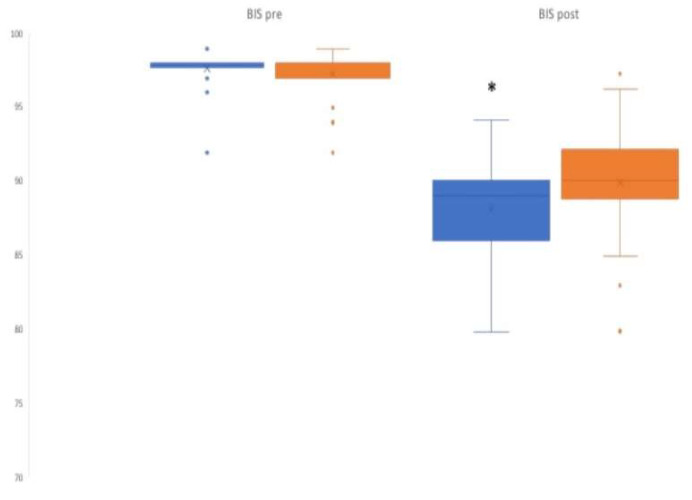
Box plots corresponding to the BIS before and after treatment. The box is delimitated by the first and the third quantiles, with the median inside. The whiskers indicate maximum and minimum values. The points represent outliers, the x represent mean values. * *p* < 0.05. Blue indicates Music Therapy Group and orange indicates Control group.

**Figure 4:**
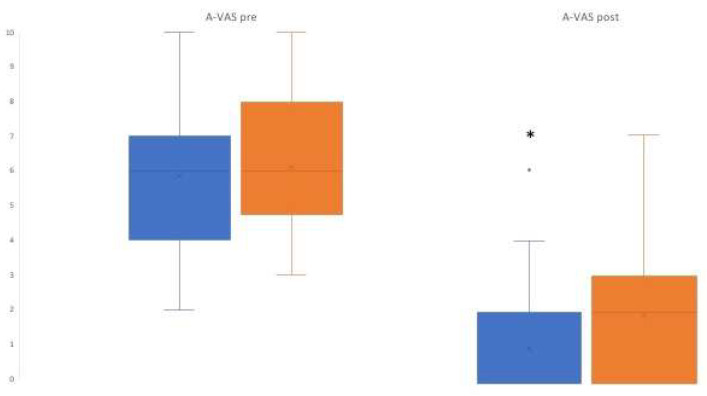
Box plots corresponding to the A-VAS before and after treatment. The box is delimitated by the first and the third quantiles, with the median inside. The whiskers indicate maximum and minimum values. The points represent outliers, the x represent mean values. * *p* < 0.05. Blue indicates Music Therapy Group and orange indicates Control group.

Box plots corresponding to the BIS and A-VAS variables before and after interventions are shown in Figure 3 and Figure 4, respectively, highlighting the decrease in the considered features, and showing in both cases that the median is significantly lower in the MTG than in the CG.

**Figure 5:**
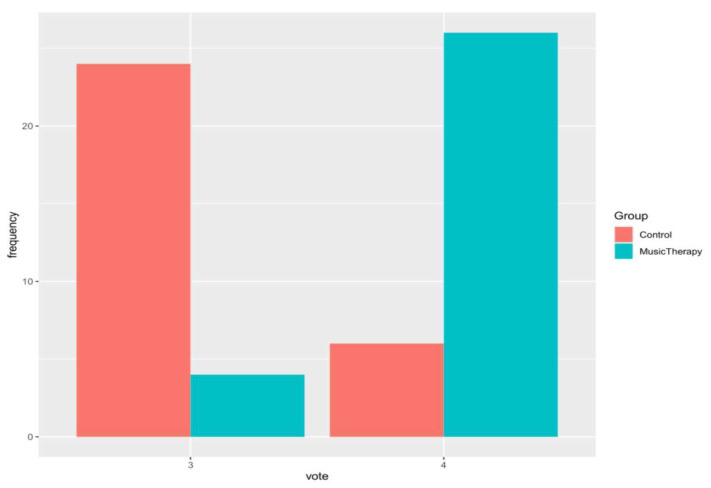
Bar plot related of patient global impression scale (PGIS); rates: 3—satisfied, and 4—very satisfied.

The patient satisfaction at the end of surgery, recorded with the PGIS, revealed high levels of satisfaction in both groups: 26 out of 30 patients (87%) in the MTG were very satisfied, and 4 out of 30 (13%) were satisfied. In the CG, 24 out of 30 patients (80%) were satisfied and 6 out of 30 (20%) were very satisfied. The satisfaction rates, evaluated using the Fisher’s test, reveal a preference for the treatment administered to the MTG rather than that given to the CG (*p* < 0.01). Figure 5 depicts the frequencies of the gradient of the two treatments.

In the MTG, 70% of patients answered that they really appreciated this experience and that MT had positively influenced their level of anxiety related to surgery. Moreover, 90% of patients in the MTG answered that they would recommend listening to music to someone who is undergoing surgery (Supplementary Figure S1).

## 4. Discussion

The present study reveals that preoperative music therapy, like intravenous midazolam, is able to promote a preoperative state of anxiolysis and sedation in patients undergoing stomatology surgery, as shown by the BIS score reduction in the MTG compared to CG.

It could also have a more pronounced long-lasting effect on surgery-related anxiety than midazolam does, as the post-surgery A-VAS reveals. No other significant change in the PAS, PAD, HR, or in the mean PRL, GH and cortisol levels between the two groups were registered. Moreover, higher levels of satisfaction were reported in the MTG.

To our knowledge, this is the first study to investigate the effect of MT compared to IV midazolam in stomatology surgery.

This research supports the body of literature that suggests MT can modulate preoperative anxiety and pain, and offer a safe and cost-effective option in standard surgical care [33], even if no study shows that MT can replace anxiolytic drugs for definite [34].

Previous studies have shown that, due to its ability to distract, music produces a physiologic state in which anxiety is likely to diminish [35].

On a neurophysiological level, anxiety activates the responses of the sympathetic system, while music stimulates the relaxation response via the activation of the parasympathetic branch of the autonomic nervous system, which leads to decreased adrenergic activity and decreased neuromuscular arousal [36].

Moreover, music modulates pain responses in different parts of the central nervous system, such as the prefrontal area, the periaqueductal gray, and the rostral ventromedial medulla, which are all parts of the descending pain modulatory system [37]. All of these areas are involved in pain, but also in depression, anxiety, and the loss of cognition, further contributing to the beneficial effect of this intervention.

Studies have demonstrated that music activates the ventral tegmental area and the nucleus accumbens, and thus leads to the release of dopamine [38], which regulates the perception of pleasure.

Music is a powerful positive stimulus that evokes and modulates both emotions and mood [39,40], and improves emotional health through coping.

A recent systematic review [41] demonstrated that the most significant impact of music administered to surgical patients is associated with a positive effect on their psychological aspects. The main result was a decrease in anxiety and a significant improvement in mood, even if the authors underlined that the response to music is unique to each person, depending on each subject’s culture, experience and musical history.

However, music listening alone may not be sufficient to reduce the anxiety and pain caused by an impending surgical procedure [42,43,44].

The present study suggests that MT has the same effect on the patient’s sympathetic activity and hormonal response as IV midazolam. We cannot postulate as to whether listening to music for a longer duration (more than five minutes) would have the same effect. Future studies are needed to verify whether adrenergic activity can be modulated by music therapy in the entire perioperative period when compared to anxiolytic drugs.

The waiting time before surgery causes as much anxiety as the surgery itself [14,32]. Due to this, in this study, patients were not subjected to passive listening to pre-recorded music provided by nurses or other medical staff. On the contrary, they were actively engaged in a relational process, together with the music and the MTp, and were facilitated to refocus their attention to the music [14,45].

This receptive MT approach prepared the patients for listening to the music through a brief guided relaxation of the breath and by focusing on an image with a positive association; this enabled a positive start to the listening process.

Music and imagery promote a temporary escape from a stressful reality, and f take the patient’s attention away from the negative stimuli present in the operating room towards something comfortable and familiar [46].

The presence of a certified MTp meant that patients were not alone with the music and enabled a tailored listening experience based on the assessment of each patient in that moment; the MTp could offer verbal instructions for refocusing, and could change or stop the music if necessary.

Furthermore, our results seem to demonstrate that 5 min of preoperative MT produces a significant reduction in alertness and a more relaxed state in a very short time [14,47], with no impact on the total length of stay of the patients in the operating room. This enables a strict standardized MT protocol to be performed even in a busy, noisy and chaotic setting, such as in the operating room.

Finally, a higher level of satisfaction in the MTG compared to the CG was reported, even if a recent publication reported that midazolam may influence postsurgical pain [48]. These results highlight that a MT intervention (with a certified music therapist) is not a case of simply listening to music, and instead allows the MTp to build an empathic relationship with the patient. With the aim of taking care of surgical patients, and not only curing them, MT can enable the perioperative procedure to be experienced in a more relaxed way.

### Limitations

The main limitations of this study are related to its design, such as the use of a small simple size and the lack of blinding and/or a placebo-controlled group, even if we have tried to overcome these limitations using standardized outcomes, such as physiological parameters and the BIS. Moreover, this trial enrolls only a specific population (i.e., patients undergoing oral surgery from a single center in Italy), therefore limiting the generalizability of the results.

Although statistically significant differences were observed in the BIS score and A-VAS between the two groups, the clinical and medical impact of these differences may be not relevant to the clinical induction of anesthesia. Therefore, the clinical implication could be that MT has the same effects on anxiety, stress and the deepness of sedation as midazolam. The main advantages of MT, as described in the present paper, include the fact that it was conducted by a specialized MTp, the lack of any adverse events and the high levels of patient satisfaction. The potential positive effect of the MTG due to the additional unilateral attention given to patients by the ancillary MTp personnel cannot be excluded. In addition, separating the impact of the MT itself from the additional impact of the MT personnel in attendance (to prevent patients from being alone) is almost impossible. On the other hand, the aim of this study was to specifically explore the effect of a specific music intervention conducted by a certified MTp, and not the effect of a “simple” process of listening to music that is not standardized nor personalized to every single patient. In our opinion, breath regulation practices and imaginative activities cannot be considered as detached from music listening, but need to be considered as one. Therefore, the reduction in anxiety should be considered as the net effect of this composite approach; this is the most important difference between music therapy and other music-based interventions in the medical setting.

Moreover, the durability of the effect of MT was not explored, even if the reduction in the postoperative A-VAS suggests that MT is able to control patients’ anxiety up to 1 h after the end of the surgical procedure.

The adoption of a MT intervention in an operative setting is surely challenging, but the present study demonstrates that a short MT session does not impact on the total length of stay of the patients in the operating room and is strongly appreciated by the patients. The costs of MT should be also considered, and this study was not designed to specifically address this issue; however, it can be argued that MT, by reducing anxiety and perioperative stress at least as well as IV drugs, but without any side effects, could be regarded as an interesting alternative. The net balance between MT and IV midazolam should be investigated in larger studies with a longer follow up period.

Finally, the potential additive or synergistic effects of the combination of MT and midazolam require further investigation, and represents an interesting area for upcoming studies, especially considering the reported effects of midazolam on postoperative pain perception [47].

## 5. Conclusions

A short, preoperative, composite and well-defined MT session could be an alternative to intravenous midazolam when aiming to induce relaxation and reduce patient anxiety during stomatology surgery. Moreover, patients in the MTG reported higher levels of satisfaction. This could result in more patient-focused care in the perioperative period. Further studies should be conducted in order to reveal whether MT may have additive effects alongside anxiolytic agents or is able to replace them, and to analyze the cost-effectiveness of this approach in the perioperative period.

## Figures and Tables

**Table 1:** Baseline characteristics.

Characteristics	Music Therapy (*n* = 30)	Control (*n* = 30)
Age (years)	61 ± 12	61 ± 12
Females, *n* (%)	28 (47.5)	26 (17.5)
ASA		
I, N (%)	4 (13.3)	3 (10)
II, *n* (%)	16 (53.4)	12 (40)
III, *n* (%)	10 (33.3)	15 (50)
Surgery		
BRONJ, *n* (%)	11 (12.5)	12 (35)
Oral cystectomy, *n* (%)	1 (45)	2 (62.5)
Carcinoma, *n* (%)	9 (20)	10 (17.5)
Others, *n* (%)	9 (15)	6 (15)
Time of surgery (min)	117 ± 22	130 ± 23

Data are expressed as mean ± SD. ASA: American Society of Anesthesiologists.

**Table 2:** Outcomes assessed before (panel a) and after (panel b) the interventions in both groups.

Outcomes	Music Therapy (*n* = 30)	Control Group (*n* = 30)	*p*-Value
(a) Pre-interventions			
Prolactin (mIU/L)	355.7 ± 504.5	239.76 ± 105.2	0.22
GH (ng/mL)	0.1 ± 1.26	1.7 ± 3.12	0.24
Cortisol (mcg/L)	13.7 ± 6	14.8 ± 6.5	0.77
SBP (mm Hg)	140.6 ± 14.6	137.7 ± 20.6	0.53
DBP (mm Hg)	77.4 ± 8.9	77.1 ± 7.1	0.85
HR (bpm)	83.9 ± 9.2	82 ± 9.4	0.79
BIS (median)	98	98	0.28
A-VAS (median)	6	6	0.67
(b) Post-interventions			
Prolactin (mIU/L)	1880 ± 1020	1730 ± 0.92	0.54
GH (ng/mL)	1.51 ± 3.93	1.10 ± 2.04	0.82
Cortisol (mcg/L)	23.1 ± 20.1	20.6 ± 18	0.77
SBP (mm Hg)	124 ± 17	122 ± 13	0.98
DBP (mm Hg)	77.4 ± 8.9	77.1 ± 7.1	0.85
HR (bpm)	75.5 ± 9.6	76.1 ± 7.7	0.77
BIS (median)	89	90	0.02 *
A-VAS (median)	0	2	0.01 *
Satisfaction score 4 (*n*, %)	26 (87%)	6 (20%)	<0.001 *

Data are expressed as mean ± SD; except for VAS and BIS. GH: growth hormone SBP: systolic blood pressure; DBP: diastolic blood pressure; HR: heart rate. BIS: bispectral index; A-VAS: anxiety visual analogic scale. Satisfaction score 4 = very satisfied, see text for details. * Two-tailed test *p*-value.

## Data Availability

The data presented in this study are available on request from the corresponding author. The data are not publicly available due to privacy issues.

## Supplementary Materials

The following supporting information can be downloaded at: https://www.mdpi.com/article/10.3390/jcm12093215/s1, Table S1. CONSORT 2010 checklist of information to include when reporting a randomised trial.

## Glossary

Music therapy	MT
Control group	CG
Music therapist	MTp
Music therapy group	MTG
Systolic blood pressure	SBP
Diastolic blood pressure	DBP
Heart rate	HR
Plasma prolactin	PRL
Growth hormone	GH
Bispectral index	BIS
Anxiety visual analogues scale	A-VAS
Patient global impression of satisfaction	PGIS
Bisphosphonate-related osteonecrosis of the jaws	BRONJI
